# 20-year trends of distal femoral, patellar, and proximal tibial fractures: a Danish nationwide cohort study of 60,823 patients

**DOI:** 10.1080/17453674.2019.1698148

**Published:** 2019-12-04

**Authors:** Veronique Vestergaard, Alma Becic Pedersen, Peter Toft Tengberg, Anders Troelsen, Henrik Morville Schrøder

**Affiliations:** a Department of Orthopaedic Surgery, Slagelse Hospital, Naestved, Slagelse and Ringsted Hospitals, Slagelse, Denmark;; b Department of Orthopaedic Surgery, Copenhagen University Hospital Hvidovre, Copenhagen, Denmark;; c The Harris Orthopaedics Laboratory, Department of Orthopaedic Surgery, Massachusetts General Hospital, Boston, Massachusetts, USA;; d Department of Clinical Epidemiology, Aarhus University Hospital, Aarhus, Denmark

## Abstract

Background and purpose — Knee fracture treatment burden remains unknown, impeding proper use of hospital resources. We examined 20-year trends in incidence rates (IRs) and patient-, fracture-, and treatment-related characteristics of knee fracture patients.

Patients and methods — This nationwide cohort study of prospectively collected data including patients with distal femoral, patellar, and proximal tibial fractures from the Danish National Patient Registry during 1998–2017, assesses IRs of knee fractures (per 10^5^ inhabitants) as well as patient-, fracture-, and treatment-related characteristics of knee fracture patients.

Results — During 1998–2017, 60,823 patients (median age 55; 57% female) sustained 74,106 knee fractures. 74% of the study population had a Charlson Comorbidity Index (CCI) of 0 and 18% a CCI of ≥ 2. 51% were proximal tibial fractures, 31% patellar fractures, and 18% distal femoral fractures. At the time of knee fracture, 20% patients had concomitant near-knee fractures (femur/tibia/fibula shaft/hip/ankle), 13% concomitant fractures (pelvic/spine/thorax/upper extremities), 5% osteoporosis, and 4% primary knee osteoarthritis. Over 1/3 knee fractures were surgically treated and of these 86% were open-reduction internal fixations, 9% external fixations, and 5% knee arthroplasties. The most common surgery type was proximal tibia plating (n = 4,868; 60% female). Knee fracture IR increased 12% to 70, females aged > 51 had the highest knee fracture IR, proximal tibial fracture had the highest knee fracture type IR (32) and surgically treated knee fracture IR increased 35% to 23.

Interpretation — Knee fracture IRs, especially of surgically treated knee fractures, are increasing and proximal tibial fracture has the highest knee fracture type IR. Females aged > 51 and patients with comorbidity are associated with knee fracture, proximal tibial fracture, proximal tibial fracture surgery, and posttraumatic knee arthroplasty.

Knee fractures include fractures in the distal femur, patella, and proximal tibia, with a reported incidence rate (IR) of approximately 9/10^5^ per year in the United States (Lambers et al. 2012). Knee fractures vary in type and complexity and often result in lower function, work performance, and health-related quality of life (Van Dreumel et al. 2015, Sluys et al. 2016). Previous studies are limited to small sample sizes, lower extremity fractures, tibial plateau fractures, patellar fractures, or proximal tibia fractures (Court-Brown and Caesar 2006, Scholes et al. 2014, Elsoe et al. 2015, Larsen et al. 2016, Wennergren et al. 2018). To our knowledge, there are no population-based studies describing IRs of knee fractures over time, either overall or according to sex, age, knee fracture type, and treatment type. Estimation of treatment burden of knee fractures for subsequent allocation of hospital resources requires knowledge of epidemiology and IRs. We conducted a national cohort study to examine 20-year trends in incidence rates (IRs) and patient-, fracture-, and treatment-related characteristics of knee fracture patients in Denmark during 1998–2017.

## Patients and methods

### Study design and data sources

The study was designed as a nationwide cohort study of prospectively collected data from the Danish Civil Registration System (CRS) and Danish National Patient Registry (DNPR) (Schmidt et al. 2014, 2015). The CRS contains complete information on Danish Civil Personal Register (CPR) number, residency, and emigration and is updated daily with vital status. The DNPR contains information on hospital admissions, emergency department visits, admission date, CPR number, age, sex, WHO ICD-10 classification, and the Danish version of the Nordic Medico-Statistical Committee Classification of Surgical Procedures (NOMESCO) (WHO 2019, NOMESCO 2019). The current NOMESCO classification was implemented in 1995; we therefore excluded the years before 1996 to reduce surgery code bias, and years 1996–1997 to exclude a potential backlog of already prevalent knee fracture cases with hospital follow-up. Data on the population were divided by sex and age for each calendar year of 1998–2017 (Statistics Denmark 2019).

### Study population

**Figure 1.**
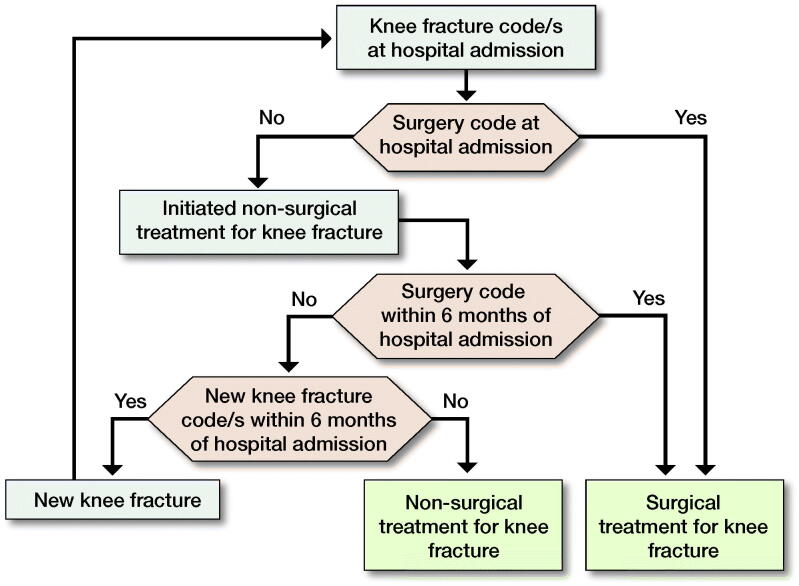
Definition of new knee fracture, surgically treated knee fracture and non-surgically treated knee fracture.

The study population consisted of patients registered in the DNPR from January 1, 1998 to December 31, 2017 with hospital contacts for ICD-10 codes DS724, DS820, and DS821 (knee fracture patients) with/without subsequent knee surgery NOMESCO code(s) (Appendix 1, see Supplementary data). Figure 1 provides a definition of new knee fracture, surgically treated knee fracture, and non-surgically treated knee fracture. Surgically treated knee fractures were divided into 3 surgery types: open reduction internal fixation (ORIF), external fixation, and knee arthroplasty. The updated version of the Charlson Comorbidity Index (CCI) was used to evaluate comorbidity (Bjorgul et al. 2010, Quan et al. 2011).

### Statistics

The study was conducted according to the Strengthening the Reporting of Observational Studies in Epidemiology (STROBE) Statement guidelines (von Elm et al. 2014). Proportions, median, and interquartile range (IQR) were used to describe the study population. We estimated annual overall IRs of knee fractures with 95% confidence intervals (CIs) as the number of knee fractures per calendar year divided by the total number of individuals at risk in Denmark in that same year. All IRs were calculated/10^5^ inhabitants. Annual IRs were calculated according to sex, age group, knee fracture type, and surgery type. Poisson regression was used to estimate incidence rate ratios (IRRs) with 1998 as year of reference. IRR expressed the relative change in IRs of knee fractures in 1999–2017 compared with year 1998 as reference IRR. All analyses were performed using the statistical software R 3.4.2 (R Foundation for Statistical Computing, Vienna, Austria).

### Ethics, registration, funding, and potential conflicts of interest

The study was approved by Danish Data Protection Agency, record number REG-085-2017. The study was funded by P. Carl Petersen Foundation, Danish Rheumatism Association, Research Unit Naestved, Slagelse and Ringsted Hospitals, Production, Research and Innovation Naestved, Slagelse and Ringsted Hospitals, Data and Development Support Region Zealand, Naestved, Slagelse and Ringsted Hospitals’ Research Fund, and Clinical Orthopaedic Research Hvidovre, Hvidovre Hospital. Funding sources were not involved in study design, collection, analysis, interpretation, and completion. The authors declare no conflicts of interest regarding this study.

## Results

### Overall incidence rates

**Figure 2.**
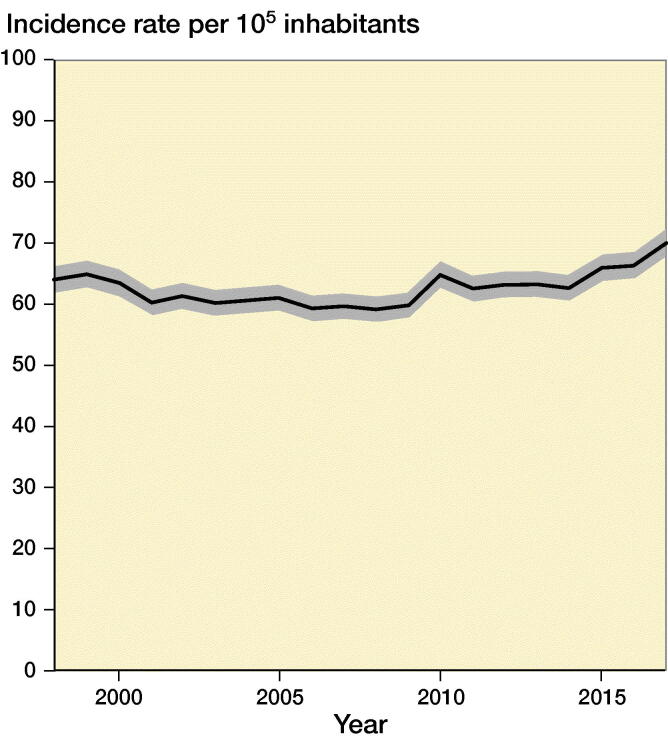
Incidence rate of knee fractures per 10^5^ inhabitants during 1998–2017 with 95% confidence intervals.

Average IR (per 10^5^ inhabitants) for sustaining knee fracture during 1998–2017 was 63 (CI 62–63). IR for knee fracture was 64 (CI 61–66) in 1998, remaining stable in the following years and increasing from year 2010 up to 70 (CI 67–72) in 2017, corresponding to a 12% increase (Figure 2). The corresponding IRR was 1.1 (CI 1.0–1.2) in 2017 compared with 1998 (Table 1, see Supplementary data).

**Table 3.** Distribution of knee fracture type in the study population including surgically and non-surgically treated knee fractures in the DNPR during 1998–2017. Values are frequency (%)

Knee fracture type	Study population n = 74,106	Surgically treated n = 24,215	Non-surgically treated n = 46,397
Proximal tibia	38,080 (51)	12,175 (50)	22,411 (48)
Patella	22,689 (31)	5,977 (24)	16,712 (36)
Distal femur	13,337 (18)	6,063 (25)	7,274 (16)

### Incidence rates by sex and age

**Figure 3.**
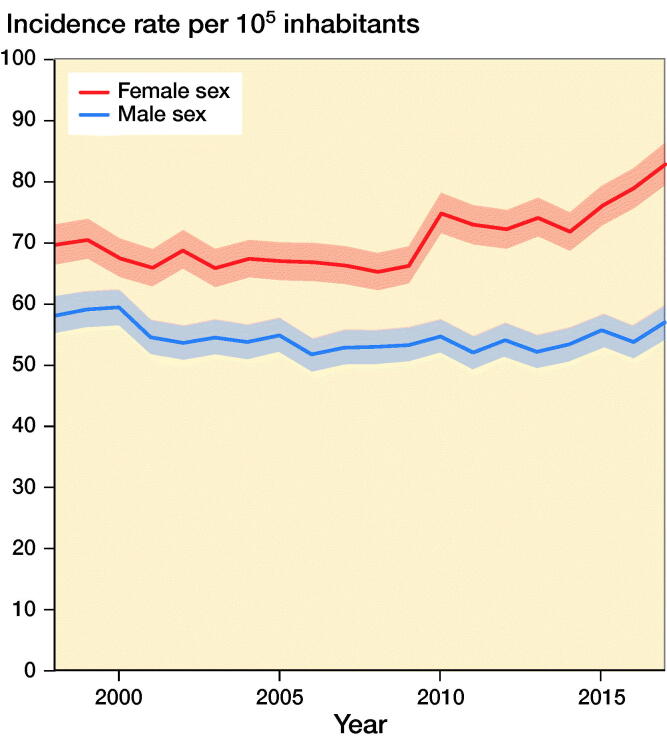
Incidence rates of knee fractures per 10^5^ inhabitants during 1998–2017 by sex.

IR of knee fractures in females increased from 70 (CI 67–73) in 1998 to 83 (CI 80–86) in 2017. IR in males decreased slightly from 58 (CI 55–61) in 1998 to 57 (CI 54–60) in 2017 (Figure 3). The corresponding IRR was 1.2 (CI 1.1–1.3) for females and 1.0 (CI 0.9–1.1) for males in 2017 compared with 1998.

During 1998–2017, IR for knee fractures was highest among females aged > 71 (IR decreased from 240 (CI 223–258) in 1998 to 223 (CI 209–239) in 2017). During 1998–2017, IR increased in females aged 51–70 years (IR increased from 95 [CI 87–103] to 124 [CI 116–132]), children aged 0–5 years (IR in females aged 0–5 years increased from 23 [CI 17–30] to 78 [CI 66–93] and IR in males aged 0–5 years increased from 24 [CI 19–32] to 71 [CI 60–84] and females and males aged 6–18 (IR in females increased from 31 [CI 26–37] to 38 [CI 33–44] and IRs in males increased from 57 [CI 50–65] to 70 [CI 63–79]).

### Incidence rates by knee fracture type

**Figure 4.**
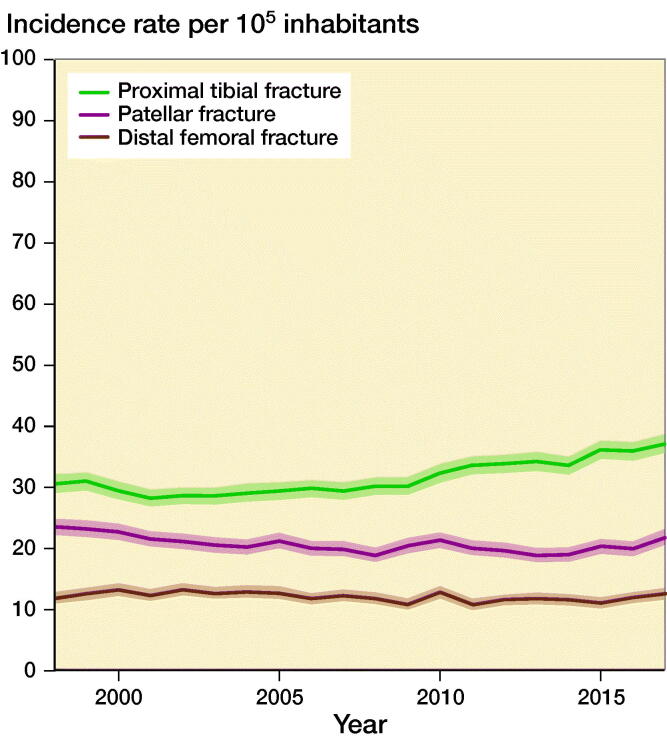
Incidence rates of knee fractures per 10^5^ inhabitants during 1998–2017 by knee fracture type.

During 1998–2017, average IR for proximal tibia fracture was 32 (CI 31–32), average IR for patella fracture was 21 (CI 21–21) and average IR for distal femur fracture was 12 (CI 12–12). IR for proximal tibia fracture increased over time, especially after 2010, whereas IRs for patella fracture and distal femur fracture remained stable (Figure 4).

### Incidence rates by treatment type

**Figure 5.**
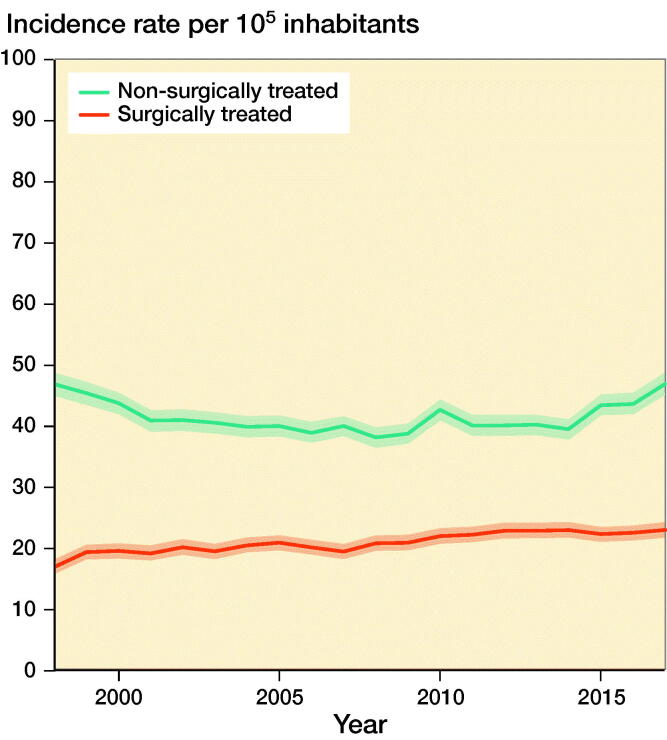
Incidence rates of surgically and non-surgically treated knee fractures per 10^5^ inhabitants during 1998–2017.

Average IR for surgically treated knee fractures was 21 (CI 21–21). IR for surgically treated knee fractures was 17 (CI 16–18) in 1998 increasing to 23 (CI 22–24) in 2017, corresponding to a 35% increase (Figure 5). The corresponding IRR for surgically treated knee fracture was 1.4 (CI 1.2–1.5) in 2017 when comparing 2017 with reference year 1998.

Average IR for non-surgically treated knee fractures was 42 (CI 41–42). IR for non-surgically treated knee fractures was 47 (CI 45–49) in 1998 and 47 (CI 45–49) in 2017, remaining stable during the 20-year period (Figure 5). The corresponding IRR for non-surgically treated knee fracture was 1.0 (CI 1.0–1.1) in 2017 when comparing 2017 with reference year 1998.

### Patient-, fracture-, and treatment-related characteristics

**Figure 6.**
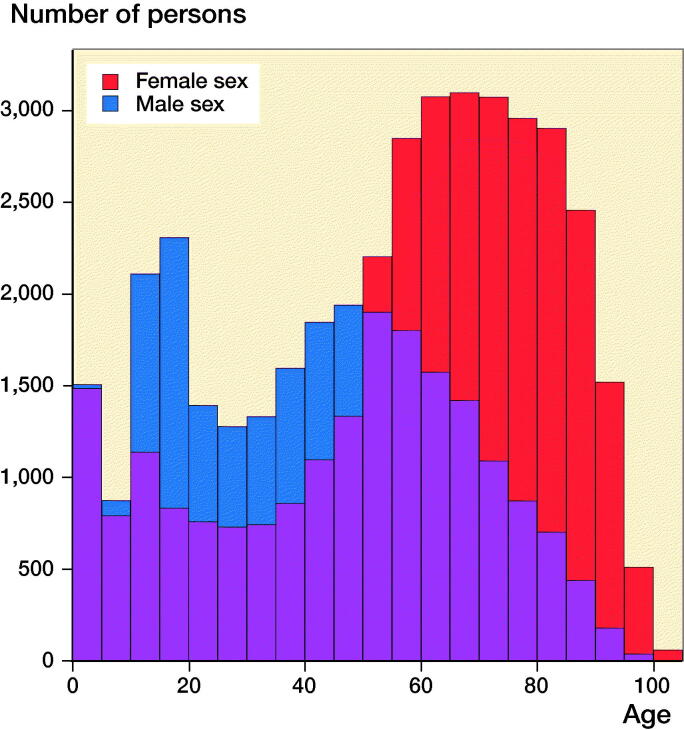
Sex and age distribution of the study population registered in the Danish National Patient Registry during 1998–2017.

During 1998–2017, 60,823 patients sustained 74,106 knee fractures (Table 2, see Supplementary data). Median study population age was 55 (IQR 30–72), being 64 years (IQR 46–78) in females and 42 years (IQR 19–59) in males. 57% of knee fracture patients were female. Children aged 0–5, males aged 5–50, and females aged > 50 had highest risk of knee fracture (Figure 6). 74% of the study population had CCI 0, 8% CCI 1, and 18% CCI ≥ 2. At the time of knee fracture, 20% of knee fracture patients were registered with concomitant near-knee fractures (femur/tibia/fibula shaft/hip/ankle), of which tibia shaft fracture (5%) and femur shaft fracture (4%) were most frequent, while 13% of knee fracture patients were registered with concomitant fractures (pelvic/spine/thorax/upper extremities) (Appendix, see Supplementary data). At the time of knee fracture, 10% of patients were registered with lesions inside the knee, 5% with osteoporosis, and 4% with primary knee osteoarthritis (OA) (Appendix, see Supplementary data).

**Table 4.** Frequency, age and sex distribution of most frequent knee fracture surgery types registered in the DNPR during 1998–2017

Knee fracture surgery type	n	Median age (IQR)	Female sex (%)
Proximal tibia plate and screws	4,868	57 (45–67)	60
Patella wiring	4,592	61 (44–72)	58
Proximal tibia screw fixation	3,635	50 (32–63)	50
Distal femur plate and screws	2,517	74 (61–85)	73
Distal femur intramedullary nail	1,350	75 (59–85)	74
Primary cemented knee arthroplasty	855	68 (59–76)	74

DNPR = Danish National Patient Registry. IQR = interquartile range.

The most common knee fracture type was proximal tibia fracture (51%), followed by patella fracture (31%) and distal femur fracture (18%). Table 3 shows distribution of knee fracture type in the study population including surgically and non-surgically treated knee fractures in the DNPR during 1998–2017. 90% of patients had 1 knee fracture registered and 11% patients had > 1 knee fracture registered. The total number of knee fracture treatments was 68,419 (some treatments covered multiple knee fractures). Of these 68,419 treatments, 34% were surgical treatments and 66% non-surgical treatments. 6% of knee fracture patients received both surgical and non-surgical treatments. 89% patients had 1 knee fracture surgery and 11% had > 1 knee fracture surgery. In non-surgically treated patients, the corresponding numbers were 96% with 1 non-surgical knee fracture treatment and 4% with > 1 non-surgical treatment.

22,996 surgeries were performed on 24,215 knee fractures in 20,350 patients during 1998–2017. In surgically treated patients, median population age was 59 years (IQR 42–72) and median age for females was 66 (IQR 54–77). Of surgically treated knee fractures, 86% surgeries were ORIFs, 9% external fixations, and 5% knee arthroplasties. Table 4 presents frequency, age, and sex distribution of the most frequent knee fracture surgery types registered in the DNPR during 1998–2017.

## Discussion

### Study limitations

Information bias, i.e., misclassifications of diagnosis codes and missing/incomplete data in variable and diagnosis registrations by discharging physicians, is present in registry studies. Fortunately, both ICD-10 and NOMESCO classifications are used to identify knee fracture patients, registering to DNPR is improving, reporting to DNPR is mandatory and is used for reimbursement by public and private hospitals (although knee fracture patients are not treated in the Danish private sector), and DNPR data quality is high with orthopedic diagnoses having the highest positive predictive value: 91% (Schmidt et al. 2015). Another study limitation is incomplete laterality registration in DNPR, likely producing overestimated IRs, but random variation cannot be excluded. The same applies for ABC extensions of ICD-10 codes: only a small percentage of the study population was registered with ABC extensions, thus distributions of knee fracture subtypes could not be described (Schmidt et al. 2015). IRs calculated at the beginning of the study period might be inflated because it is unknown if they are incidences or a backlog of prevalent knee fractures with hospital follow-up. Nevertheless, the possible error in average IR for the period decreases over the long 20-year study period. The overestimations are also reduced by the 6-month wash-out period (Figure 1). The 6-month wash-out period is likely not too short because only 11% patients had > 1 knee fracture registered. The study was a 20-year, nationwide cohort study of knee fracture patients from a relatively homogenous population, providing results of high external validity.

### Overall incidence rates

The knee fracture IR was approximately 9/10^5^ per year in the United States (Lambers et al. 2012). The IR was ∼60/10^5^ inhabitants in our study (Figure 2). However, the American study included only data from emergency department visits in a population sample.

### Incidence rates by sex, age, knee fracture type, and treatment type

In our study, increasing knee fracture IRs were seen in females aged > 51 and > 71 and both sexes aged 0–18. A national Danish study of fracture incidences reported increases in IRs of lower extremity fractures in males aged < 50, females aged > 50, and both sexes aged > 75 (Driessen et al. 2016). This was a 1-year study and did not discriminate between proximal tibia and distal femur fractures; it can therefore only partly be extrapolated to our results.

A British single-center study calculated IRs/10^5^ person-years at risk (PYRS) for distal femoral, patellar, and proximal tibial fractures in patients aged > 65 (Court-Brown et al. 2014). Their IRs ranged between 8 and 37 compared with our IRs of 12–32/10^5^ inhabitants. As in our study, older patient groups had high knee fracture IRs, especially older females, and proximal tibia had a high knee fracture type IR.

Our demographic data accord with a Swedish tibial fracture study in which proximal tibia fracture patients were more likely to be females (58%) with higher mean age (54) (Wennergren et al. 2018). The average IR for proximal tibia fracture was 27/10^5^ PYRS, which is comparable to our results: 32/10^5^ inhabitants. Our results mirror the results of the Swedish study regarding increase in proximal tibia fracture IR, higher IRs of females with increasing age compared with males, and more flatlined IR knee fracture curves in males.

A Danish tibial plateau fracture study demonstrated that intra-articular proximal tibia fractures present a treatment burden, showing increased tibial plateau fracture IRs in males aged < 50 and females aged > 50 (Elsoe et al. 2015), which echoes our results of higher IRs in proximal tibia fractures and higher knee fracture IRs in females aged > 51.

Driessen et al. (2016) calculated an annual patella fracture IR in Denmark: 33/10^5^ PYRS. Larsen et al. (2016) studied patella fractures where IRs varied between 11 and 17/10^5^ PYRS and females aged 60–80 had the highest patella fracture IR. Our results show a similar trend in high patella fracture IR (21) and that the highest knee fracture IRs are seen in females aged > 51 and > 71. Remaining discrepancies in results can be explained by different study period lengths and in geographical differences.

Improvement in registrations to the DNPR, increased societal demand for invasive orthopedic treatments, i.e., surgical treatments of knee fractures and broader inclusion of comorbidly challenged patients with a lower threshold for knee fracture surgery, might explain the 35% increase in surgically treated knee fracture IR. Most patients (89%) had only 1 knee fracture surgery, making double registrations an unlikely contributor to IR increase.

### Patient-, fracture-, and treatment-related characteristics of knee fracture patients

At the time of knee fracture, 18% patients had CCI ≥ 2, 1/5 concomitant near-knee fractures, 13% concomitant fractures, 5% osteoporosis, and 4% primary knee OA. Females aged > 51 and patients with comorbidity are associated with sustaining knee fracture, especially proximal tibial fracture, surgical treatment for knee fracture, proximal tibial fracture surgery, and posttraumatic TKA (Table 4), making our results similar to trends described in current literature with knee fractures and TKA procedures being most frequent in older females (Court-Brown and Caesar 2006, Court-Brown et al. 2014, Kremers et al. 2014, Elsoe et al. 2015, Larsen et al. 2016, Driessen et al. 2016, Krause et al. 2016, Wennergren et al. 2018).

## Conclusion

In this 20-year nationwide cohort study, we observed that overall IR of knee fracture increased 12% to 70/10^5^ inhabitants while IR of surgically treated knee fracture increased 35% to 23/10^5^ inhabitants. Our findings reflect the complexity of the knee fracture population with future challenges concerning treatment burden, increasing incidences, and patient risk groups and provide the basis for proper hospital resource allocations including computing future risk-adjustment and payment models.

## Supplementary data

Appendix and Tables 1–2 are available as supplementary data in the online version of this article, http://dx.doi.org/10.1080/17453674.2019.1698148.

## Supplementary Material

Supplemental Material
