# Supplementary material for: 20-year trends of distal femoral, patellar, and proximal tibial fractures: a Danish nationwide cohort study of 60,823 patients
Source: Acta Orthop. 2019 Dec 4;91(1):109–14. doi: 10.1080/17453674.2019.1698148 (PMC7006734; doi:10.1080/17453674.2019.1698148)
Supplement: Supplemental Material [file IORT_A_1698148_SM1128.pdf]

Supplementary data

Appendix

ICD-10 diagnosis codes

Concomitant near-knee fractures: DS720 (femoral neck fracture), DS721 (pertrochanteric femoral fracture), DS722 (subtrochanteric femoral fracture), DS723 (femoral shaft fracture), DS727 (multiple femoral fractures), DS728 (other femoral fracture), DS729 (femoral fracture, other, not otherwise specified), DS822 (tibial shaft fracture), DS823 (distal tibia fracture), DS824 (fibula shaft fracture), DS825 (medial malleolar fracture), DS826 (lateral malleolar fracture), DS827 (multiple fractures of the knee and lower knee), DS828 (fracture of other part of lower leg) and DS829 (fracture of lower leg, other, not otherwise specified).

Concomitant fractures: DS122 (fracture of cervical spine), DS127 (multiple fractures of cervical spine), DS22 (fracture of costae, sternum or thoracic spine), DS32 (fracture of lumbar spine or pelvis), DS42 (shoulder and/or upper arm fracture), DS52 (elbow and/or lower arm fracture), DS62 (wrist and/or hand fracture), DT021 (fractures in thorax, lumbar spine and pelvis), DT027 (fractures in thorax, lumbar spine, pelvis, upper and lower extremities) and DT08 (fracture of spine, not otherwise specified).

Lesions inside the knee: DS83 (luxation and distortion of joint and ligaments in knee).

Primary knee osteoarthritis (OA): DM170 (primary bilateral knee OA), DM171 (primary unilateral knee OA) and DM179 (knee OA other, not otherwise specified).

Osteoporosis: DM81 (osteoporosis without pathological fracture).

Proximal tibial fracture: DS821.

Patellar fracture: DS820.

Distal femoral fracture: DS724.

Femoral shaft fracture: DS723.

Tibial shaft fracture: DS822.

NOMESCO surgery codes

External fixation codes: KNFJ25 (external fixation of fracture in distal femur), KNGJ20 (external fixation of fracture in patella), KNGJ21 (external fixation of fracture in proximal tibia) and KNGJ29 (external fixation of fracture in knee, not otherwise specified).

Knee arthroplasty codes: KNGB (primary prosthesis in the knee), KNGC (secondary prosthesis in the knee) and KNGG (joint resections, arthroplasties and arthrodeses in knee joint).

Open reduction internal fixation (ORIF), external fixation and reduction codes:

Distal femur:

KNFJ0 closed reduction of femur fracture
KNFJ05 closed reduction of fracture in distal femur
KNFJ1 open reduction of femur fracture
KNFJ15 open reduction of fracture in distal femur
KNFJ2 external fixation of femur fracture
KNFJ25 external fixation of fracture in distal femur
KNFJ3 internal fixation with bio implants of femur fracture
KNFJ35 internal fixation with bio implants of fracture in distal femur
KNFJ4 internal fixation with wires, rods, cerclage wiring or pins/needles of femur fracture
KNFJ45 internal fixation with wires, rods, cerclage wiring or pins/needles of fracture in distal femur
KNFJ5 internal fixation with intramedullary nail of femur fracture
KNFJ55 internal fixation with intramedullary nail of fracture in distal femur
KNFJ6 internal fixation with plate and screws of femur fracture
KNFJ65 internal fixation with plate and screws of fracture in distal femur
KNFJ7 internal fixation with screws only of femur fracture
KNFJ75 internal fixation with screws only of fracture in distal femur
KNFJ8 internal fixation with other or combined method of fracture in femur
KNFJ85 internal fixation with other or combined method of fracture in distal femur
KNFJ9 other surgical fracture treatment in femur
KNFJ95 other surgical fracture treatment in distal femur
KNGF2 fixation of articular surface fragment in knee joint

Patella/proximal tibia:

KNGJ fracture treatment in knee and lower leg
KNGJ0 closed reduction of fracture in knee
KNGJ00 closed reduction of fracture in patella
KNGJ01 closed reduction of fracture in proximal tibia
KNGJ09 closed reduction of fracture in knee, not otherwise specified
KNGJ1 open reduction of fracture in knee
KNGJ10 open reduction of fracture in patella
KNGJ11 open reduction of fracture in proximal tibia
KNGJ19 open reduction of fracture in knee, not otherwise specified

KNGJ2 external fixation of fracture in knee
KNGJ20 external fixation of fracture in patella
KNGJ21 external fixation of fracture in proximal tibia
KNGJ29 external fixation of fracture in knee, not otherwise specified
KNGJ3 internal fixation with bio implants of fracture in knee
KNGJ30 internal fixation with bio implants of fracture in patella
KNGJ31 internal fixation with bio implants of fracture in proximal tibia
KNGJ39 internal fixation with bio implants of fracture in knee, not otherwise specified
KNGJ4 internal fixation with wires, rods, cerclage wiring or pins/needles of fracture in knee
KNGJ40 internal fixation with wires, rods, cerclage wiring or pins/needles of fracture in patella
KNGJ41 internal fixation with wires, rods, cerclage wiring or pins/needles of fracture in proximal tibia
KNGJ49 internal fixation with wires, rods, cerclage wiring or pins/needles of fracture in knee, not otherwise specified
KNGJ5 internal fixation with intramedullary nail of fracture in knee
KNGJ51 internal fixation with intramedullary nail of fracture in proximal tibia
KNGJ59 internal fixation with intramedullary nail of fracture in knee, not otherwise specified
KNGJ6 internal fixation with plate and screws of fracture in knee
KNGJ60 internal fixation with plate and screws of fracture in patella
KNGJ61 internal fixation with plate and screws of fracture in proximal tibia
KNGJ69 internal fixation with plate and screws of fracture in knee, not otherwise specified
KNGJ7 internal fixation with screws only of fracture in knee
KNGJ70 internal fixation with screws only of fracture in patella
KNGJ71 internal fixation with screws only of fracture in proximal tibia
KNGJ79 internal fixation with screws only of fracture in knee, not otherwise specified
KNGJ8 internal fixation with other or combined method of fracture in knee
KNGJ80 internal fixation with other or combined method of fracture in patella
KNGJ81 internal fixation with other or combined method of fracture in proximal tibia
KNGJ89 internal fixation with other or combined method of fracture in knee, not otherwise specified
KNGJ9 other surgical fracture treatment in knee
KNGJ90 other surgical fracture treatment in patella
KNGJ91 other surgical fracture treatment in proximal tibia
KNGJ99 other surgical fracture treatment in knee, not otherwise specified

Table 1. Annual incidence rates per 10^5 inhabitants with 95% confidence intervals (IR (CI)) and incidence rate ratios (IRR (CI)) for the study population registered in the DNPR during 1998–2017

Year	IR (CI)	IRR (CI)
1998	64.0 (61.9–66.2)	reference
1999	64.9 (62.8–67.1)	1.01 (0.97–1.06)
2000	63.5 (61.4–65.6)	0.99 (0.94–1.04)
2001	60.3 (58.2–62.4)	0.94 (0.90–0.99)
2002	61.3 (59.3–63.5)	0.96 (0.91–1.00)
2003	60.2 (58.2–62.3)	0.94 (0.90–0.99)
2004	60.6 (58.6–62.8)	0.95 (0.90–0.99)
2005	61.0 (59.0–63.2)	0.95 (0.91–1.00)
2006	59.3 (57.3–61.4)	0.93 (0.88–0.97)
2007	59.6 (57.6–61.7)	0.93 (0.89–0.98)
2008	59.2 (57.1–61.2)	0.92 (0.88–0.97)
2009	59.8 (57.8–61.9)	0.93 (0.89–0.98)
2010	64.8 (62.8–67.0)	1.01 (0.97–1.06)
2011	62.5 (60.5–64.6)	0.97 (0.93–1.02)
2012	63.2 (61.1–65.3)	0.99 (0.94–1.03)
2013	63.3 (61.2–65.4)	0.99 (0.94–1.04)
2014	62.6 (60.6–64.8)	0.98 (0.93–1.03)
2015	65.9 (63.8–68.1)	1.03 (0.98–1.08)
2016	66.4 (64.3–68.5)	1.04 (0.99–1.09)
2017	70.0 (67.9–72.2)	1.09 (1.04–1.15)

DNPR = Danish National Patient Registry.

Table 2. Demographic data on the study population (N = 60,823) registered in the DNPR during 1998–2017. Values are frequency (%) unless otherwise specified

Patient demographics	Year 1998	1999–2004	2005–2010	2011–2016	2017
Patients, total no.	3,262	17,623	17,557	18,870	3,511
Age, median (IQR)	54 (33–73)	55 (33–74)	55 (30–72)	56 (27–71)	57 (27–72)
Female sex	1,800 (55)	9,804 (56)	9,875 (56)	11,012 (58)	2,073 (59)
Charlson co-morbidity index					
Zero (CCI = 0)	2,710 (83)	13,729 (78)	12,784 (73)	13,303 (71)	2,491 (71)
Low (CCI = 1)	169 (5)	1,186 (7)	1,482 (8)	1,788 (10)	355 (10)
Medium (CCI = 2)	229 (7)	1,601 (9)	1,757 (10)	2,056 (11)	368 (11)
High (CCI ≥ 3)	154 (5)	1,107 (6)	1,534 (9)	1,723 (9)	297 (9)
Other diagnoses					
Concomitant near-knee fractures	666 (20)	3,970 (23)	3,763 (21)	3,293 (18)	448 (13)
Concomitant fractures	390 (12)	2,314 (13)	2,355 (13)	2,259 (12)	269 (8)
Lesions inside the knee	303 (9)	1,767 (10)	1,826 (10)	2,034 (11)	315 (9)
Primary knee OA	116 (4)	789 (5)	786 (5)	708 (4)	63 (2)
Osteoporosis	76 (2)	571 (3)	879 (5)	1,128 (6)	102 (3)
Knee fractures, total no.	3,487	20,391	20,257	22,133	4,104
Knee fracture type					
Proximal tibia	1,613 (46)	9,342 (46)	9,643 (48)	11,622 (53)	2,126 (52)
Patella	1,242 (36)	6,913 (34)	6,653 (33)	6,627 (30)	1,254 (31)
Distal femur	632 (18)	4,136 (20)	3,961 (20)	3,884 (18)	724 (18)
Inhabitants at risk [a]	5,220,478				5,722,369

DNPR = Danish National Patient Registry. IQR = interquartile range.
[a] Total number of inhabitants at risk in the specific calendar period.
